# A test of somatic mosaicism in the androgen receptor gene of Canada lynx (*Lynx canadensis*)

**DOI:** 10.1186/s12863-015-0284-y

**Published:** 2015-10-26

**Authors:** Melanie B. Prentice, Jeff Bowman, Paul J. Wilson

**Affiliations:** Department of Environmental & Life Sciences, Trent University, 1600 West Bank Drive, Peterborough, K9J 7B8 ON Canada; Wildlife Research and Monitoring Section, Ontario Ministry of Natural Resources and Forestry, 2140 East Bank Drive, Peterborough, K9J 7B8 ON Canada; Biology Department, Trent University, 1600 West Bank Drive, Peterborough, K9J 7B8 ON Canada

**Keywords:** Somatic mosaicism, Androgen receptor, Canada lynx, Trinucleotide repeats

## Abstract

**Background:**

The *androgen receptor*, an X-linked gene, has been widely studied in human populations because it contains highly polymorphic trinucleotide repeat motifs that have been associated with a number of adverse human health and behavioral effects. A previous study on the *androgen receptor* gene in carnivores reported somatic mosaicism in the tissues of a number of species including Eurasian lynx (*Lynx lynx*). We investigated this claim in a closely related species, Canada lynx (*Lynx canadensis*). The presence of somatic mosaicism in lynx tissues could have implications for the future study of exonic trinucleotide repeats in landscape genomic studies, in which the accurate reporting of genotypes would be highly problematic.

**Methods:**

To determine whether mosaicism occurs in Canada lynx, two lynx individuals were sampled for a variety of tissue types (lynx 1) and tissue locations (lynx 1 and 2), and 1,672 individuals of known sex were genotyped to further rule out mosaicism.

**Results:**

We found no evidence of mosaicism in tissues from the two necropsied individuals, or any of our genotyped samples.

**Conclusions:**

Our results indicate that mosaicism does not manifest in Canada lynx. Therefore, the use of hide samples for further work involving trinucleotide repeat polymorphisms in Canada lynx is warranted.

**Electronic supplementary material:**

The online version of this article (doi:10.1186/s12863-015-0284-y) contains supplementary material, which is available to authorized users.

## Background

The X-linked *androgen receptor* (*AR*) gene codes for a transcription factor that controls the binding of androgens in different tissue types [1–3]. The organization and location of the *AR* gene on the X-chromosome has been conserved for both male and female placental, marsupial and monotreme mammals [3, 4]. Androgenic hormones including testosterone and dihydrotestosterone are integral in a number of bodily processes, most notably sexual differentiation and development [5]. The wide range of functions that the *AR* gene encompasses has concurrently lead to a range of disease-associated phenotypes, which have been linked to variable tandem trinucleotide repeats occurring in the first codon of the *AR* gene coding sequence [6]. Trinucleotide repeats are repeat structures that consist of units that are 3 nucleotides long, caused by the selection against frame-shift mutations which would alter the reading frame of the transcribed protein [7]. The natural variation of these repeats within humans indicates that these motifs have a critical role in “normal” protein function and evolutionary adaptation [8, 9]. More specifically, trinucleotide repeats are known to affect phenotype, such that disease in humans has been attributed to frequency of repeats exceeding a certain threshold, beyond which, the transcriptional activity of the *AR* gene is affected [10, 11]. For this reason, trinucleotide repeat fragments of the *AR* gene have been extensively studied in humans for their potential role in infertility [12, 13], aggressive or dominant behavior [14–16], criminal activity [17, 18], personality disorders [19, 20], and the development of some cancers and other diseases [21–25].

Studies of the *AR* gene in wildlife are rare but are likely to become more frequent in the future as the role of trinucleotide markers in mediating adaptive evolution in contemporarily short time-frames becomes more clear [26]. While it is well understood that climate change will have profound effects on wildlife [27], we are currently unable to predict whether species will be able to adapt and evolve new strategies to cope with the increasing environmental change. The characterization of exonic standing genetic variability will therefore allow for a better understanding of the adaptive capacities of populations to be resilient to the effects of stressful events including climate change. As a result, there is a recognized need to identify and characterize the genetic variability of fitness-related traits [28] and the response of genes to environmental change [29, 30]. Trinucleotide repeats are particularly desirable candidates for studies of the genomics of adaptation because they occur in as many as 20 % of human genes, have relatively higher rates of mutation than single nucleotide polymorphisms (SNPs), and can show consistently high levels of within-population variation [6, 26]. Importantly, such high rates of mutation may facilitate adaptation to stressors (e.g., climate change) in contemporarily short timeframes. Recently, several studies have demonstrated the potential evolutionary and adaptive importance of trinucleotide repeats within clock genes in both birds [31] and fish [32]. Thus, the study of trinucleotide repeat structures in a range of other vertebrate species [8, 26, 33, 34] offers the potential to use the properties of microsatellite repeats [35] to understand the genomics of rapid adaptation.

Historically, the characterization of the *AR* gene has been affected by biological and technical issues, with implications for accurate genotyping. More specifically, somatic mutations and allele peak morphology issues have been encountered upon scoring size separated alleles differing in the number of exonic trinucleotide repeats [36–38]. Mosaicism in biological systems can be defined as “the presence of more than one genetically distinct cell line in a single organism” in which tissue-to-tissue genetic variations occur that may not follow Mendelian rules of inheritance ([39]; p. 748). More recently, Köhler et al. (2005) [p. 106] describe somatic mosaicism as “different proportions of cells containing either mutant or wild-type proteins that are present in various tissues of the same individual [22]”. Telenius et al. (1994) provided the first report of heterogenic somatic mosaicism of CAG repeats in tissues [40]. Since then, several studies have detected tissue-specific somatic mosaicism of CAG repeats in the *AR* gene in both the neural and non-neural tissues of individuals with Huntington’s disease, spinal bulbar muscular atrophy, spinocerebellar ataxia type 1, denatorubural-pallidoluysian atrophy and Machado-Joseph disease [21]. For individuals with androgen insensitivity syndrome, genotype-phenotype discrepancies have been traced to somatic mosaicism of the *AR* gene itself [36, 37].

Much of the research conducted on the *AR* gene to date has involved the study of human disease. Trinucleotide repeats in the *AR* gene have yet to be correlated with transcriptional activity in species other than humans, and the limited number of studies that have been conducted on other species suggests lower levels of variability than in humans [41, 42]. Of particular interest is a study by Wang et al. (2012) who examined the variability of *AR* trinucleotide repeat in carnivores through sequencing of the first exon in the *AR* gene (containing three trinucleotide repeat tracts) [42]. The authors reported a change in CAG repeat number in the same tissues of a number of carnivore species, indicating tissue-specific mosaicism patterns in the *AR* gene of studied species. In their study, somatic mosaicism was evident in all three poly-glutamine tracts within exon 1of the *AR* gene, with a maximum extent of five alleles in several carnivore species. The authors concluded that the higher frequency of tissue-specific mosaicism in the *AR* gene of carnivores compared to other studied taxa implies that carnivores tend to exhibit mosaicism [42].

The objective of our study was to test for somatic mosaicism in a carnivore, the Canada lynx (*Lynx canadensis*). Canada lynx are closely related to the Eurasian lynx (*Lynx lynx*), one of the species shown by Wang et al. (2012) to exhibit somatic mosaicism. We consider it important to evaluate the potential for somatic mosaicism in Canada lynx before conducting further research on the *AR* gene. If allelic patterns of mosaicism are revealed, simple genotyping of individuals may not provide conclusive results with respect to genetic variability of individuals at this gene, which could complicate high throughput genotyping of individuals at the *AR* gene. Further, if mosaicism in this gene is caused by trinucleotide repeat instabilities, there will be important consequences for future studies that wish to examine trinucleotide repeat variability in wildlife species at any gene. This makes the investigation of potential somatic mutations a worthwhile goal as somatic mosaicism could significantly confound the use of trinucleotide repeat markers in the study of the adaptive genomics of wildlife. In such a case, we will need to begin considering the more dynamic nature of genes within genomes when designing studies, in particular those containing trinucleotide repeats.

We test the hypothesis that somatic mosaicism occurs in the androgen receptor gene in Canada lynx. We report *AR* genotypes for multiple samples taken from two necropsied lynx, as well as hide samples from lynx sampled at multiple locations across Canada.

## Methods

To address the question of whether or not Canada lynx exhibit mosaicism at the *AR* gene, we designed a study that was composed of two levels of analysis. First, we conducted necropsies and tissue sampling of two lynx individuals (one full carcass and one hide), which allowed for multiple samples of various tissue types to be taken from one individual and a variety of sampling locations spanning the entire lynx carcasses in both individuals. Second, as we recognize that the sample size from the necropsies alone is limited, we genotyped additional samples collected across the Canada lynx range to verify our findings on a broader scale. Canada lynx are currently listed as not at risk by the Committee on the Status of Endangered Wildlife in Canada (COSEWIC), and are legally harvested annually. Thus, we obtained our additional samples either through licensed, commercial fur harvest, or under the authority of the Ontario Ministry of Natural Resources and Forestry (OMNRF). While sequence data would provide additional information about repeat purity (i.e., perfect vs. imperfect repeat structures) and the potential for SNPs within the flanking regions of the repeats, we conducted microsatellite genotyping on all of our samples as mosaicism can very easily be detected as size based variants. Mosaicism was evident in [42] largely based on size, indicating that if mosaicism is present in our study species, we should be able to detect it given our study used the same primers as [42] in addition to our large sample size.

### Necropsy sampling

To test the hypothesis that somatic mosaicism exists in Canada lynx tissues, a necropsy was conducted for strategic sampling of two lynx individuals. The first individual (lynx 1) consisted of an entire carcass and the second (lynx 2) was a hide. The lynx carcass was a road-killed individual that was collected by the Ontario Ministry of Natural Resources and Forestry in 2010 and stored frozen until tissue sampling was conducted to ensure optimal preservation of high-quality tissues for DNA extraction. The lynx hide was collected in 2006 from a fur harvester in Ontario, Canada. It was important for the purpose of assessing the influence of the *AR* gene in different tissues, to obtain and analyze the genetic profile of a large number of different cell types. A total of 87 hide, muscle, liver and brain samples were taken from the two individuals. The liver we sampled had five lobes; two main lobes rested on top of three smaller lobes.

### DNA extraction, quantification and amplification

DNA extraction and quantification was solely performed on the necropsy samples. DNA for the remaining 1,672 lynx samples (979 males and 693 females) was previously extracted from hide tissue according to the protocols outlined in [43], and was available in working concentration for PCR amplification. The availability of hide tissues from both museum specimens and fur auction houses makes this tissue type highly accessible for the genetic surveying of Canada lynx and other furbearer populations (e.g., [44–47]). The hide samples in our study represent individuals trapped in Yukon, British Columbia, Alberta, Manitoba, Ontario, and Quebec, Canada, as well as Alaska, USA.

Tissues were prepared for extraction by mincing approximately 1 mm X 1 mm pieces of tissue and placing it in 500ul of 1X lysis buffer [4 M Urea, 0.2 M NaCl, 0.5 % n-lauroyl sarcosine, 10 mM 1,2-cyclohexanediaminetetraacatic acid (CDTA), 0.1 M Tris–HCl (pH 8) and 600 U/ml proteinase K (Roche Applied Science, Laval QC)]. DNA from tissues was extracted by a modified version of the MagneSil® (Promega) manufacturers protocol, in which 200ul of the prepared tissues was substituted for the suggested 60ul of whole blood, and the number of wash steps was reduced [48]. All liquid handling was carried out by a JANUS® Automated Workstation from Perkin Elmer. Extracted DNA was quantified by PicoGreen® (Invitrogen) method according to the manufacturers protocols [49, 50].

From quantification, samples were normalized to a working concentration of 2.5 ng/ul and amplified with the primers developed by [42], which capture a ~700 bp region of exon 1 containing three trinucleotide repeat tracts. Amplification was conducted in a 10ul reaction containing deionized water (Invitrogen), 1X PCR Reaction Buffer (Invitrogen), 2 mM MgCl_2_ (Invitrogen), 0.2 mM dNTP solution (Invitrogen), 0.2 mg/mL BSA, 0.4uM forward and reverse primers (forward primer labeled with the fluorescent dye HEX) (Integrated DNA Technologies), 0.025U Invitrogen Platinum Taq DNA Polymerase, and 5 ng of DNA. The PCR reaction was run in a Bio-Rad DNA Engine Dyad and Dyad Disciple thermocycler under the following conditions: 95 °C for 10 min; followed by 29 cycles of 94 °C for 30 s, 58 °C for 1 min, and 72 °C for 1 min, and completed with a step of 65 °C for 15 min.

**Fig. 1:**
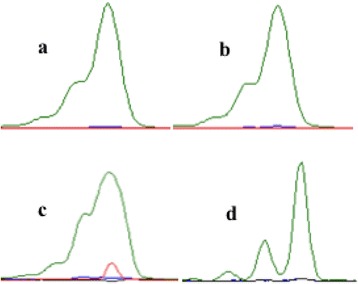
Differential peak morphologies of *androgen receptor* alleles resulting from DNA dilution and reagent use. Lynx positive control DNA sample amplified with Invitrogen Taq DNA Polymerase and diluted to 1:10 (**a**), 1:20 (**b**), and 1:50 (**c**) ratios with deionized water. Lynx positive control DNA sample amplified with Invitrogen Platinum Taq DNA Polymerase (no dilution necessary) (**d**)

Difficulties and biases in PCR amplification have been previously reported for the *AR* gene (e.g., [38]), most likely due to the high GC content in many exonic trinucleotide repeat fragments including *AR*. Many researchers have since obtained successful amplification and improved results by substituting Invitrogen Platinum Taq DNA Polymerase for the standard Invitrogen Taq DNA Polymerase (e.g., [51]). Such improvements were also evident in our study (Fig. 1).

### Sexing of lynx necropsy individuals

The knowledge of sex for each individual allowed for the development of a search image for detecting mosaicism. For male lynx tissues, a homozygous genotype is expected as the *AR* gene is X-linked, and males should therefore only inherit a single copy of the gene. In our study, any heterozygote male individual is a candidate for exhibiting mosaicism. Female lynx can be homozygous or heterozygous at the *AR* gene naturally, however, the allelic diversity of lynx at this locus predicts three allele patterns should be observed if mosaicism is occurring. If mosaicism were detected in female individuals with three alleles, the extent of mosaicism in females would still be an underestimate given that heterozygous females could be undetected somatic homozygous individuals. In the necropsy analysis, mosaicism would be suggested if more than the expected number of alleles were discovered across multiple samples from the same individual (i.e., more than one allele for males and two alleles for females across all samples).

To confirm sex of necropsied lynx, two samples from each individual (one hide and one muscle from Lynx 1 and two hide samples from Lynx 2) were amplified at two sex loci. The first primer pair, SRY-Y53-3D-F and SRY-Y53-3C-R amplified a ~218 bp region of the SRY genetic marker [52]. The second locus, a ~447 bp region of the ZFX/ZFY genetic marker, was amplified with the primer pair ZFX-P3-3EZ-F and ZFX-P3-5EZ-R [53]. Amplification was conducted in a 10ul reaction containing deionized water (Invitrogen), 10X PCR Reaction Buffer (Invitrogen), 50 mM MgCl_2_ (Invitrogen), 100 mM dNTP solution (Invitrogen), 3 mg/mL BSA, 40uM forward and reverse primers (Integrated DNA Technologies) mentioned above (forward primers labeled with the fluorescent dye HEX), 0.0375U Invitrogen Taq DNA Polymerase, and 5 ng of DNA. The PCR reaction was run in a Bio-Rad DNA Engine Dyad and Dyad Disciple thermocycler under the following conditions: 94 °C for 15 min; followed by 29 cycles of 94 °C for 30 s, 52 °C for 1 min 30 s, and 72 °C for 1 min 30 s, and completed with a step of 60 °C for 45 min. Amplified samples were run on an 80 mL, 1.5 % agarose gel stained with ethidium bromide at 90 volts for 45 min, and visualized under ultraviolet light and to determine sex. Female individuals were identified by the presence of two bands, and males, by the presence of three bands on the gel. Controls of a known male and female lynx were included to rule out technological errors and strengthen conclusions.

### Genotyping

For genotyping, 5ul of MapMarker 1000 X-Rhodamine (MM-1000-Rox) size standard (BioVentures) was mixed into 1 mL of deionized HiDi Formamide (Applied Biosystems), and 9.5ul of this product was added to 0.5ul of each amplified sample. Genotyping was performed on the Applied Biosystems 3730 DNA Analyzer. Genotypes were scored with SoftGenetics LLC GeneMarker AFLP/Genotyping Software Version 1.91. We used GenAlEx version 6.5 (Peakall & Smouse 2006, 2012) to calculate allele and genotype frequencies for both males and females.

## Results & discussion

We observed ten different alleles across all genotypes samples, ranging between sizes 711–744 bp (including flanking sequence). The smallest three alleles observed were only found in a single female individual each, and no individuals with alleles 717 or 723 within the allelic range were found. The most common alleles were observed in the middle of the allelic range (Tables 1 and 2).

**Table 1:** Allele frequencies of the trinucleotide repeat tracts within exon 1 of the *androgen receptor* (*AR*) gene in Canada lynx (*Lynx canadensis*)

Allele	Frequency	Frequency	Frequency
	(Males only)	(Females only)	(All samples)
711	0.000	0.001	0.000
714	0.000	0.001	0.000
720	0.000	0.001	0.000
726	0.109	0.078	0.096
**729**	**0.401**	**0.379**	**0.392**
**732**	**0.289**	**0.335**	**0.313**
735	0.100	0.119	0.108
738	0.077	0.075	0.076
741	0.007	0.009	0.008
744	0.008	0.003	0.006

Frequencies are shown for male samples only (*N* = 979), female samples only (*N* = 693), and both males and females combined (all samples; *N* = 1672). As the *AR* gene is X-linked, and all males are therefore homozygous, allele frequencies are equivalent to genotype frequencies for males. No individuals were observed with alleles 717 or 723. The two most common alleles are in bold

**Table 2:** Genotype frequencies of the trinucleotide repeat tract within exon 1 of the *androgen receptor* (*AR*) gene in Canada lynx (*Lynx canadensis*). Frequencies are shown for female samples only (*N* = 693)

First allele/Second allele	711	714	720	726	729	732	735	738	741	744
711	-	-	-	-	-	-	-	-	-	-
714	0.001	-	-	-	-	-	-	-	-	-
720	-	-	-	-	-	-	-	-	-	-
726	-	-	-	0.009	-	-	-	-	-	-
729	-	-	-	0.059	0.160	-	-	-	-	-
732	-	-	-	0.039	0.253	0.123	-	-	-	-
735	-	-	-	0.019	0.078	0.081	0.020	-	-	-
738	-	-	0.001	0.017	0.040	0.048	0.017	0.012	-	-
741	-	-	-	0.004	0.006	0.004	0.001	0.001	-	-
744	-	-	-	-	0.001	0.001	0.001	0.001	-	-

Sex identification indicated that the necropsied lynx represented one female (lynx 1) and one male (lynx 2) specimen. Of the tissues analyzed at the *AR* gene from these individuals (62 from lynx 1 and 25 from lynx 2), all resulted in a single clear genotype for each individual (a consistent homozygote and heterozygote genotype across all tissue samples for the male and female, respectively).

Additional genotyping of the 1,672 lynx samples did not detect somatic mosaicism in any of our male or female Canada lynx samples, although a single sample was removed from the data set due to contamination (see Additional file 1). All other samples fell within our search image of what is expected in a typical individual not exhibiting mosaicism (all males were homozygotes and no females exhibited more than two alleles). The absence of any evidence of mosaicism in Canada lynx does not provide conclusive evidence that it is not present in other, unanalyzed individuals, however, given the high allelic diversity of the *AR* gene in Canada lynx, if undetected, mosaicism would still only be present at a negligible level due to the large sample size we surveyed. For the purposes of our study, the overall lack of detection, coupled with our large sample size indicates that mosaic events do not pose a high risk of confounding large-scale analyses and genotyping in this study system, nor is an important biological mechanism within Canada lynx.

Our findings are inconsistent with those of Wang et al. (2012) who found *AR* mosaicism in multiple carnivore species [42]. It is possible that expression of the somatic mutation causing *AR* mosaicism is absent in Canada lynx in particular, but does manifest in Eurasian lynx and other carnivore tissues at a higher rate. As we evaluated a large sample of lynx hides, we suggest that lynx hide tissue can be used to study the *AR* gene in Canada lynx without the risk of issues caused by mosaicism.

## Conclusions

The implications of somatic mosaicism within exonic trinucleotide repeat polymorphisms can have important influences on the accurate reporting and use of genotypes in studies of landscape genomics. This potential issue, however, is rarely considered in research outside of human disease studies. As the role of exonic repeat fragments in mediating adaptive evolution becomes clearer, it is likely that the prevalence of their use in wildlife genomic studies will increase. This makes the evaluation of somatic mosaicism in these repeat fragments imperative. In this study, we report no evidence of mosaicism in our two necropsied lynx individuals, or our larger screening of Canada lynx hide tissue. All males were homozygous for a single allele, and there was no evidence of more than two alleles in females, which would have been predicted if mosaicism was present given the allelic diversity of the gene in lynx. Our results indicate that even if mosaicism is present in this species, its prevalence is low given our inability to detect mosaicism in our large sample size. Therefore, the use of hide samples for further work involving trinucleotide repeat polymorphisms in Canada lynx is warranted, given that the *AR* gene appears to follow typical patterns of a X-linked gene in this species.

## Availability of data

Genotypic data supporting the findings of this study can be found on the Dryad Digital Repository: http://dx/10.5061/dryad.h43c1.

## Additional file

Additional file 1:
**During the course of the work for this manuscript, a single male lynx was identified as heterozygous at the** ***AR*** **gene.** A quantitative analysis was conducted on this sample to evaluate possible alternative hypotheses including; somatic mosaicism, chromosomal abnormalities (e.g., an XXY male sample) and sample contamination. Information on this analysis and its results are contained within the supplementary information document associated with this manuscript. (DOCX 16 kb)

## Abbreviations

AR: Androgen receptor
SNPs: Single nucleotide polymorphisms
COSEWIC: Committee on the Status of Endangered Wildlife in Canada
OMNRF: Ontario Ministry of Natural Resources and Forestry
