# Supplementary material for: A test of somatic mosaicism in the androgen receptor gene of Canada lynx (Lynx canadensis)
Source: BMC Genet. 2015 Oct 26;16:125. doi: 10.1186/s12863-015-0284-y (PMC4623281; doi:10.1186/s12863-015-0284-y)
Supplement: Additional file 1: — During the course of the work for this manuscript, a single male lynx was identified as heterozygous at the AR gene. A quantitative analysis was conducted on this sample to evaluate possible alternative hypotheses including; somatic mosaicism, chromosomal abnormalities (e.g., an XXY male sample) and sample contamination. Information on this analysis and its results are contained within the supplementary information document associated with this manuscript. (DOCX 16 kb) [file 12863_2015_284_MOESM1_ESM.docx]

**Additional file 1**

In the course of this study, we detected a single male individual that was heterozygous at the *AR* gene. These results would have potentially indicated a mosaic individual, however they were equally consistent with the possibility of chromosomal abnormalities. To rule out either of these possibilities we conducted a quantitative analysis of the X and Y fragments in this sample. The signal ratios we observed for these fragments were inconsistent with both the mosaicism and chromosomal abnormality hypotheses. Rather, the results suggested that the male sample in question was most likely to be low-level contamination of a female sample. We also investigated 14 microsatellite loci in this sample to further screen for signs of contamination, and were able to confidently conclude that the patterns we observed were the result of low-level contamination of our sample rather than *AR* specific mosaicism. Thus, we removed the individual from our sample set and concluded that we did not detect any evidence of mosaicism in our study of Canada lynx.
